# *N*′-(2-Meth­oxy­benzyl­idene)aceto­hydrazide

**DOI:** 10.1107/S1600536810038092

**Published:** 2010-09-30

**Authors:** Tie-Ming Yu, Lu-Ping Lv

**Affiliations:** a Linjiang College, Hangzhou Vocational and Technical College, Hangzhou 310018, People’s Republic of China

## Abstract

In the title mol­ecule, C_10_H_12_N_2_O_2_, the acetohydrazide group is almost planar [within 0.0306 (12) Å] and forms a dihedral angle of 12.15 (14)° with the benzene ring. The meth­oxy group deviates from the attached benzene ring with a C—O—C—C torsion angle of 4.2 (4)°·The mol­ecule adopts a *trans* configuration with respect to the C=N bond. In the crystal, mol­ecules are linked into centrosymmetric dimers by pairs of N—H⋯O hydrogen bonds and intermolecular C—H⋯O interactions further stabilize the structure.

## Related literature

For general background to Schiff bases, see: Cimerman *et al.* (1997 ▶); Offe *et al.* (1952 ▶); Richardson *et al.* (1988 ▶). For related structures, see: Li & Jian (2008 ▶); Tamboura *et al.* (2009 ▶).
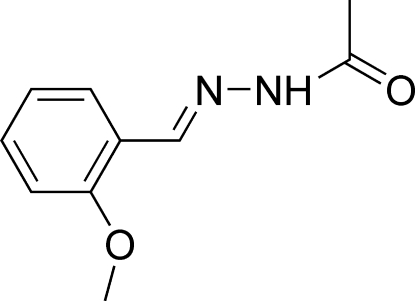


## Experimental

### 

#### Crystal data

C_10_H_12_N_2_O_2_
*M* *_r_* = 192.22
Triclinic, 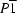

*a* = 5.3865 (7) Å
*b* = 8.4609 (11) Å
*c* = 11.3301 (14) Å
α = 77.499 (4)°
β = 76.516 (5)°
γ = 89.101 (5)°
*V* = 489.90 (11) Å^3^
*Z* = 2
Mo *K*α radiation
μ = 0.09 mm^−1^
*T* = 223 K
0.19 × 0.17 × 0.15 mm

#### Data collection

Bruker SMART CCD area-detector diffractometer
Absorption correction: multi-scan (*SADABS*; Bruker, 2002 ▶) *T*_min_ = 0.982, *T*_max_ = 0.985
4827 measured reflections
1694 independent reflections
1489 reflections with *I* > 2σ(*I*)
*R*_int_ = 0.018

#### Refinement

*R*[*F* ^2^ > 2σ(*F* ^2^)] = 0.056
*wR*(*F* ^2^) = 0.163
*S* = 1.06
1694 reflections
128 parameters
H-atom parameters constrained
Δρ_max_ = 0.40 e Å^−3^
Δρ_min_ = −0.33 e Å^−3^

### 

Data collection: *SMART* (Bruker, 2002 ▶); cell refinement: *SAINT* (Bruker, 2002 ▶); data reduction: *SAINT*; program(s) used to solve structure: *SHELXS97* (Sheldrick, 2008 ▶); program(s) used to refine structure: *SHELXL97* (Sheldrick, 2008 ▶); molecular graphics: *SHELXTL* (Sheldrick, 2008 ▶); software used to prepare material for publication: *SHELXTL*.

## Supplementary Material

Crystal structure: contains datablocks I, global. DOI: 10.1107/S1600536810038092/gw2092sup1.cif

Structure factors: contains datablocks I. DOI: 10.1107/S1600536810038092/gw2092Isup2.hkl

Additional supplementary materials:  crystallographic information; 3D view; checkCIF report

## Figures and Tables

**Table 1:** Hydrogen-bond geometry (Å, °)

*D*—H⋯*A*	*D*—H	H⋯*A*	*D*⋯*A*	*D*—H⋯*A*
N2—H2⋯O2^i^	0.86	2.07	2.899 (2)	163
C6—H6*A*⋯O1^ii^	0.96	2.59	3.491 (3)	157

Symmetry codes: (i) 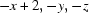; (ii) 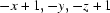.

**Fig. 1.**
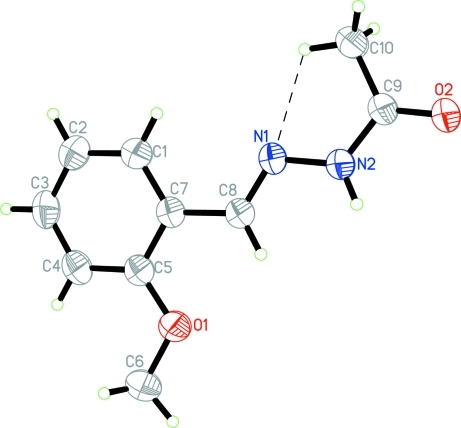
The molecular structure of the title compound. Displacement ellipsoids are drawn at the 50% probability level.

**Fig. 2.**
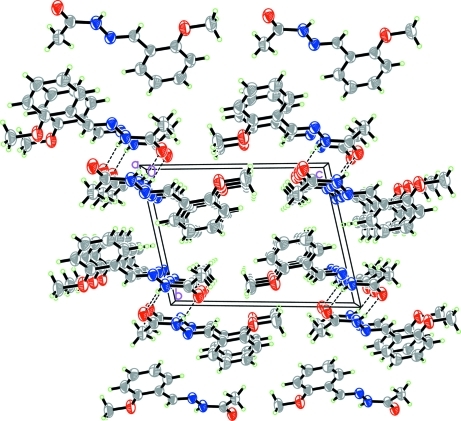
Part of the crystal packing of the title compound. Hydrogen bonds are shown as dashed lines.

## supplementary crystallographic information

### Comment

Schiff bases have attracted much attention due to the possibility of their analytical applications (Cimerman *et al.*, 1997). They are also important ligands, which have been reported to have mild bacteriostatic activity and are used as potential oral iron-chelating drugs for genetic disorders such as thalassemia (Offe *et al.*, 1952; Richardson *et al.*, 1988). Metal complexes based on Schiff bases have received considerable attention because they can be utilized as model compounds of active centres in various complexes (Tamboura *et al.*, 2009). We report here the crystal structure of the title compound (Fig. 1).

The acetohydrazide group is planar and it forms a dihedral angle of 12.15 (14)° with the benzene ring.  deviates from the attached benzene ring by 4.2 (4)°. [C6—O1—C5—C4 = 4.2 (4)°]. The molecule adopts a *trans* configuration with respect to the C═N bond. Bond lengths and angles are comparable to those observed for *N*'-[1-(4-methoxyphenyl)ethylidene]acetohydrazide (Li & Jian, 2008).

The molecules are linked by N—H···O hydrogen bonds into a centrosymmetric dimer. In addition, an intramolecular C—H···N hydrogen bond is observed.

### Experimental

2-Methoxybenzaldehyde (1.36 g, 0.01 mol) and acetohydrazide (0.74 g, 0.01 mol) were dissolved in stirred methanol (25 ml) and left for 2.5 h at room temperature. The resulting solid was filtered off and recrystallized from ethanol to give the title compound in 88% yield. Single crystals suitable for X-ray analysis were obtained by slow evaporation of an ethanol solution at room temperature (m.p. 465–467 K).

### Refinement

H atoms were positioned geometrically (N-H =  0.86 Å and C-H = 0.93 or 0.96 Å) and refined using a riding model, with *U*_iso_(H) = 1.2*U*_eq_(C,N) and 1.5*U*_eq_(C_methyl_). A rotating group model was used for the methyl groups.

### Crystal data

**Table:**

C_10_H_12_N_2_O_2_	*Z* = 2
*M**_r_* = 192.22	*F*(000) = 204
Triclinic, *P*1	*D*_x_ = 1.303 Mg m^−^^3^
Hall symbol: -P 1	Mo *K*α radiation, λ = 0.71073 Å
*a* = 5.3865 (7) Å	Cell parameters from 1694 reflections
*b* = 8.4609 (11) Å	θ = 1.9–25.0°
*c* = 11.3301 (14) Å	µ = 0.09 mm^−^^1^
α = 77.499 (4)°	*T* = 223 K
β = 76.516 (5)°	Block, colourless
γ = 89.101 (5)°	0.19 × 0.17 × 0.15 mm
*V* = 489.90 (11) Å^3^	

### Data collection

**Table:**

Bruker SMART CCD area-detector diffractometer	1694 independent reflections
Radiation source: fine-focus sealed tube	1489 reflections with *I* > 2σ(*I*)
graphite	*R*_int_ = 0.018
φ and ω scans	θ_max_ = 25.0°, θ_min_ = 1.9°
Absorption correction: multi-scan (*SADABS*; Bruker, 2002)	*h* = −6→6
*T*_min_ = 0.982, *T*_max_ = 0.985	*k* = −10→9
4827 measured reflections	*l* = −13→13

### Refinement

**Table:**

Refinement on *F*^2^	Primary atom site location: structure-invariant direct methods
Least-squares matrix: full	Secondary atom site location: difference Fourier map
*R*[*F*^2^ > 2σ(*F*^2^)] = 0.056	Hydrogen site location: inferred from neighbouring sites
*wR*(*F*^2^) = 0.163	H-atom parameters constrained
*S* = 1.06	*w* = 1/[σ^2^(*F*_o_^2^) + (0.0812*P*)^2^ + 0.265*P*] where *P* = (*F*_o_^2^ + 2*F*_c_^2^)/3
1694 reflections	(Δ/σ)_max_ < 0.001
128 parameters	Δρ_max_ = 0.40 e Å^−^^3^
0 restraints	Δρ_min_ = −0.33 e Å^−^^3^

### Special details

**Table:**

Geometry. All esds (except the esd in the dihedral angle between two l.s. planes) are estimated using the full covariance matrix. The cell esds are taken into account individually in the estimation of esds in distances, angles and torsion angles; correlations between esds in cell parameters are only used when they are defined by crystal symmetry. An approximate (isotropic) treatment of cell esds is used for estimating esds involving l.s. planes.
Refinement. Refinement of F^2^ against ALL reflections. The weighted R-factor wR and goodness of fit S are based on F^2^, conventional R-factors R are based on F, with F set to zero for negative F^2^. The threshold expression of F^2^ > 2sigma(F^2^) is used only for calculating R-factors(gt) etc. and is not relevant to the choice of reflections for refinement. R-factors based on F^2^ are statistically about twice as large as those based on F, and R- factors based on ALL data will be even larger.

### Fractional atomic coordinates and isotropic or equivalent isotropic displacement parameters (Å^2^)

**Table:**

	*x*	*y*	*z*	*U*_iso_*/*U*_eq_	
O1	0.3030 (3)	0.1576 (2)	0.41553 (14)	0.0617 (5)	
C10	0.6401 (4)	0.2210 (3)	−0.2060 (2)	0.0533 (6)	
H10A	0.5000	0.2686	−0.1579	0.080*	
H10B	0.5753	0.1429	−0.2424	0.080*	
H10C	0.7390	0.3041	−0.2707	0.080*	
N1	0.5212 (3)	0.2232 (2)	0.04470 (16)	0.0430 (5)	
N2	0.7383 (3)	0.1459 (2)	−0.00176 (16)	0.0461 (5)	
H2	0.8319	0.1014	0.0474	0.055*	
C1	0.1048 (5)	0.3982 (3)	0.1500 (2)	0.0522 (6)	
H1	0.1575	0.4195	0.0637	0.063*	
C2	−0.1084 (5)	0.4724 (3)	0.2049 (2)	0.0576 (6)	
H2A	−0.1987	0.5420	0.1560	0.069*	
C3	−0.1852 (5)	0.4423 (3)	0.3321 (2)	0.0572 (6)	
H3	−0.3285	0.4917	0.3696	0.069*	
C4	−0.0514 (5)	0.3388 (3)	0.4053 (2)	0.0562 (6)	
H4	−0.1048	0.3197	0.4915	0.067*	
C5	0.1626 (4)	0.2634 (3)	0.3506 (2)	0.0462 (5)	
C6	0.2348 (6)	0.1311 (4)	0.5483 (2)	0.0720 (8)	
H6A	0.3527	0.0593	0.5823	0.108*	
H6B	0.0651	0.0835	0.5791	0.108*	
H6C	0.2408	0.2326	0.5725	0.108*	
C7	0.2427 (4)	0.2924 (3)	0.22085 (19)	0.0418 (5)	
C8	0.4680 (4)	0.2133 (3)	0.16151 (19)	0.0421 (5)	
H8	0.5719	0.1559	0.2096	0.050*	
C9	0.8059 (4)	0.1389 (3)	−0.12303 (19)	0.0424 (5)	
O2	0.9982 (3)	0.0679 (2)	−0.16141 (14)	0.0528 (5)	

### Atomic displacement parameters (Å^2^)

**Table:**

	*U*^11^	*U*^22^	*U*^33^	*U*^12^	*U*^13^	*U*^23^
O1	0.0686 (11)	0.0756 (12)	0.0348 (8)	0.0287 (9)	−0.0062 (7)	−0.0076 (8)
C10	0.0497 (13)	0.0723 (16)	0.0378 (12)	0.0158 (11)	−0.0097 (10)	−0.0134 (11)
N1	0.0414 (9)	0.0489 (11)	0.0373 (9)	0.0120 (7)	−0.0048 (7)	−0.0116 (8)
N2	0.0431 (10)	0.0589 (12)	0.0350 (9)	0.0181 (8)	−0.0073 (7)	−0.0107 (8)
C1	0.0573 (13)	0.0584 (14)	0.0412 (12)	0.0157 (11)	−0.0123 (10)	−0.0118 (10)
C2	0.0562 (14)	0.0611 (15)	0.0585 (15)	0.0213 (11)	−0.0178 (11)	−0.0166 (12)
C3	0.0508 (13)	0.0610 (15)	0.0602 (15)	0.0174 (11)	−0.0046 (11)	−0.0242 (12)
C4	0.0582 (14)	0.0650 (15)	0.0409 (12)	0.0131 (11)	0.0008 (10)	−0.0162 (11)
C5	0.0486 (12)	0.0489 (12)	0.0391 (11)	0.0084 (9)	−0.0070 (9)	−0.0098 (9)
C6	0.094 (2)	0.083 (2)	0.0359 (13)	0.0288 (16)	−0.0138 (13)	−0.0109 (12)
C7	0.0422 (11)	0.0440 (12)	0.0387 (11)	0.0064 (9)	−0.0070 (9)	−0.0114 (9)
C8	0.0447 (11)	0.0444 (11)	0.0360 (11)	0.0088 (9)	−0.0078 (9)	−0.0088 (9)
C9	0.0394 (11)	0.0494 (12)	0.0357 (11)	0.0065 (9)	−0.0039 (8)	−0.0091 (9)
O2	0.0479 (9)	0.0689 (11)	0.0396 (8)	0.0221 (7)	−0.0051 (7)	−0.0143 (7)

### Geometric parameters (Å, °)

**Table:**

O1—C5	1.367 (3)	C2—C3	1.371 (3)
O1—C6	1.430 (3)	C2—H2A	0.9300
C10—C9	1.501 (3)	C3—C4	1.385 (4)
C10—H10A	0.9600	C3—H3	0.9300
C10—H10B	0.9600	C4—C5	1.391 (3)
C10—H10C	0.9600	C4—H4	0.9300
N1—C8	1.272 (3)	C5—C7	1.399 (3)
N1—N2	1.382 (2)	C6—H6A	0.9600
N2—C9	1.351 (3)	C6—H6B	0.9600
N2—H2	0.8600	C6—H6C	0.9600
C1—C2	1.383 (3)	C7—C8	1.470 (3)
C1—C7	1.394 (3)	C8—H8	0.9300
C1—H1	0.9300	C9—O2	1.227 (2)
			
C5—O1—C6	117.66 (19)	C3—C4—H4	119.8
C9—C10—H10A	109.5	C5—C4—H4	119.8
C9—C10—H10B	109.5	O1—C5—C4	124.3 (2)
H10A—C10—H10B	109.5	O1—C5—C7	116.03 (19)
C9—C10—H10C	109.5	C4—C5—C7	119.7 (2)
H10A—C10—H10C	109.5	O1—C6—H6A	109.5
H10B—C10—H10C	109.5	O1—C6—H6B	109.5
C8—N1—N2	115.56 (18)	H6A—C6—H6B	109.5
C9—N2—N1	121.13 (17)	O1—C6—H6C	109.5
C9—N2—H2	119.4	H6A—C6—H6C	109.5
N1—N2—H2	119.4	H6B—C6—H6C	109.5
C2—C1—C7	121.7 (2)	C1—C7—C5	118.4 (2)
C2—C1—H1	119.2	C1—C7—C8	121.2 (2)
C7—C1—H1	119.2	C5—C7—C8	120.42 (19)
C3—C2—C1	119.2 (2)	N1—C8—C7	120.22 (19)
C3—C2—H2A	120.4	N1—C8—H8	119.9
C1—C2—H2A	120.4	C7—C8—H8	119.9
C2—C3—C4	120.6 (2)	O2—C9—N2	119.71 (19)
C2—C3—H3	119.7	O2—C9—C10	122.64 (19)
C4—C3—H3	119.7	N2—C9—C10	117.65 (18)
C3—C4—C5	120.4 (2)		
			
C8—N1—N2—C9	175.77 (19)	O1—C5—C7—C1	179.4 (2)
C7—C1—C2—C3	0.5 (4)	C4—C5—C7—C1	0.5 (4)
C1—C2—C3—C4	0.0 (4)	O1—C5—C7—C8	−0.9 (3)
C2—C3—C4—C5	−0.3 (4)	C4—C5—C7—C8	−179.8 (2)
C6—O1—C5—C4	−4.2 (4)	N2—N1—C8—C7	179.40 (17)
C6—O1—C5—C7	176.9 (2)	C1—C7—C8—N1	−9.4 (3)
C3—C4—C5—O1	−178.8 (2)	C5—C7—C8—N1	171.0 (2)
C3—C4—C5—C7	0.0 (4)	N1—N2—C9—O2	−179.23 (19)
C2—C1—C7—C5	−0.8 (4)	N1—N2—C9—C10	1.3 (3)
C2—C1—C7—C8	179.6 (2)		

### Hydrogen-bond geometry (Å, °)

**Table:**

*D*—H···*A*	*D*—H	H···*A*	*D*···*A*	*D*—H···*A*
C10—H10A···N1	0.96	2.27	2.766 (3)	111
N2—H2···O2^i^	0.86	2.07	2.899 (2)	163
C6—H6A···O1^ii^	0.96	2.59	3.491 (3)	157

Symmetry codes: (i) −*x*+2, −*y*, −*z*; (ii) −*x*+1, −*y*, −*z*+1.
